# Association of hemoglobin concentration and mortality in critically ill patients with severe traumatic brain injury

**DOI:** 10.1186/cc11431

**Published:** 2012-07-20

**Authors:** Mypinder S Sekhon, Nielson McLean, William R Henderson, Dean R Chittock, Donald EG Griesdale

**Affiliations:** 1 Department of Medicine, Division of Critical Care Medicine, Vancouver General Hospital, University of British Columbia, 855 West 12th Avenue, Vancouver, BC, V5Z 1M9, Canada; 2 Department of Anesthesia, Pharmacology and Therapeutics and Department of Medicine, Division of Critical Care Medicine, Vancouver General Hospital, University of British Columbia, 855 West 12th Avenue, Vancouver, BC, V5Z 1M9, Canada; 3 Centre for Clinical Epidemiology and Evaluation, Vancouver Coastal Health Research Institute, 828 West 10th Avenue, Vancouver, BC, V5Z 1M9, Canada

## Abstract

**Introduction:**

The critical care management of traumatic brain injury focuses on preventing secondary ischemic injury. Cerebral oxygen delivery is dependent upon the cerebral perfusion pressure and the oxygen content of blood, which is principally determined by hemoglobin. Despite its importance to the cerebral oxygen delivery, the precise hemoglobin concentration to provide adequate oxygen delivery to injured neuronal tissue in TBI patients is controversial with limited evidence to provide transfusion thresholds.

**Methods:**

We conducted a retrospective cohort study of severe TBI patients, investigating the association between mean 7-day hemoglobin concentration and hospital mortality. Demographic, physiologic, intensive care interventions, clinical outcomes and daily hemoglobin concentrations were recorded for all patients. Patients were all cared for at a tertiary, level 1 trauma center in a mixed medical and surgical intensive unit. Patients were divided into quartiles based on their mean 7-day hemoglobin concentration: < 90 g/L, 90 - 99 g/L, 100 - 109 g/L and > 110 g/L. Multivariable log-binomial regression was used to model the association between mean daily hemoglobin concentration and hospital mortality.

**Results:**

Two hundred seventy-three patients with traumatic brain injury were identified and 169 were included in the analysis based on inclusion/exclusion criteria. Of these, 77% of the patients were male, with a mean age of 38 (SD 17) years and a median best GCS of 6 (IQR 5 - 7). One hundred fifteen patients (68%) received a red blood cell (RBC) transfusion. In RBCs administered in the ICU, the median pre-transfusion hemoglobin was 79 g/L (IQR 73 - 85). Thirty-seven patients (22%) died in hospital. Multivariable analysis revealed that mean 7-day hemoglobin concentration < 90 g/L was independently associated with an increased risk of hospital mortality (RR 3.1, 95% CI 1.5 - 6.3, p = 0.03). Other variables associated with increased mortality on multivariable regression were insertion of external ventricular drain, age and decreased GCS. Red blood cell transfusion was not associated with mortality following multivariable adjustment.

**Conclusions:**

A mean 7-day hemoglobin concentration of < 90g/L is associated with increased hospital mortality in patients with severe traumatic brain injury.

## Introduction

Traumatic brain injury (TBI) affects 1.4 million individuals and accounts for greater than 50,000 deaths annually in the US [1]. It is a major cause of mortality and morbidity worldwide, but in recent years, advances resulting in improved outcomes for patients with a TBI have been made in critical care [2-5]. Despite these advances, questions about the non-operative critical care management of TBI, particularly transfusion practices, remain unanswered.

Preventing secondary ischemic injury to neuronal tissue by ensuring adequate cerebral oxygen delivery is a cornerstone of post-TBI resuscitation [6-8], and the management of anemia is paramount in this process. Following TBI, up to 46% of patients have a hemoglobin of less than 90 g/L during the first week of admission, and 76% of these patients require a red blood cell (RBC) transfusion [9]. Transfusion of RBCs has been shown to increase oxygen delivery and brain tissue oxygenation [10]. However, these benefits in patients with a TBI must be weighed against established and potentially harmful effects of RBC transfusion [11-15].

Transfusion practices in the critically ill have been studied extensively [16,17]. Currently, a hemoglobin concentration of 70 g/L is an accepted transfusion trigger in an overall population of non-bleeding critically ill patients [16,17]. However, the clinical literature examining transfusion thresholds in patients with a TBI is weak and results are conflicting [18]. Both nadir hemoglobin [19] and anemia (hemoglobin of less than 90 g/L) [9] have been associated with increased mortality following TBI. In contrast, a subgroup analysis of the Transfusion Requirements in Critical Care (TRICC) trial, though underpowered, found no difference in mortality between the restrictive (70 to 90 g/L) and the liberal (100 to 120 g/L) transfusion groups for the TBI patients enrolled in the trial [16]. Not surprisingly, no specific recommendation for optimal hemoglobin thresholds in the Brain Trauma Foundation Guidelines remains [6]. Although the Lund concept recommends maintaining a hemoglobin concentration of between 125 and 140 g/L, no substantive evidence supporting this threshold remains [20]. Given this uncertainty, we performed a retrospective cohort study to determine the association of mean 7-day hemoglobin concentration and mortality in patients with a severe TBI in an academic trauma center.

## Materials and methods

The Clinical Research Ethics Board at the University of British Columbia and the Vancouver Coastal Health Authority approved the protocol and waived the requirement for written informed consent.

### Patient inclusion and data collection

A database of patients admitted to the intensive care unit (ICU) at Vancouver General Hospital (VGH) between May 2000 and March 2006 was used to construct a retrospective cohort of all patients with a severe TBI (Glasgow Coma Scale (GCS) score of not more than 8). All patients were treated in a closed, 27-bed, mixed medical-surgical unit affiliated with the University of British Columbia. The ICU at VGH operates on an approximate 1:1.2 nurse-to-patient ratio. Patients were excluded if they were obeying commands within 12 hours (indicating a non-severe TBI) or died within 12 hours of admission, as these patients were likely to succumb irrespectively of hemoglobin level. Patients were also excluded if they had a non-traumatic etiology of their decreased level of consciousness (for example, alcohol intoxication or aneurysmal subarachnoid hemorrhage) or concomitant traumatic quadriparesis. Data were collected by using standardized forms and included type and severity of injury, ICU management (including packed RBCs administered), and neuromonitoring information. Type and severity of injury were characterized by the following: baseline vital signs, best GCS score within 12 hours, mechanism and type of TBI (diffuse axonal injury, parenchymal contusions, extra-axial collections, or basal skull or calvarial fractures), surgical procedures performed, and features suggestive of increased intracranial pressure (ICP) on computerized tomography scan from radiology reports (mixed density lesion of greater than 25 mm, midline shift of greater than 5 mm, and cisternal compression).

TBI management recorded included use of bolus mannitol in the emergency room (dose of 1 g/kg), therapeutic hypothermia (target temperature and duration of therapy), and use of neuromuscular blockers. Monitoring data acquired included use of external ventricular drain (EVD), ICP measurements (if EVD was present), use of jugular venous oximetry (SjO_2_), SjO_2 _measurements, and daily GCS score measurement. Demographic and mortality data were obtained from the ICU database. We used all-cause hospital mortality as our primary endpoint. In addition, the ICU database contained the Acute Physiology and Chronic Health Evaluation II (APACHE II) score, baseline admission laboratory values, and vital signs for the first 24 hours. Daily morning hemoglobin levels were recorded for each patient. The number of units of packed RBCs administered for the first 14 days of ICU admission was also recorded.

### Traumatic brain injury management

Since 1995, VGH has used a TBI management protocol that is based on the Brain Trauma Foundation Guidelines [6]. All patients are maintained with the head of bed at at least 30 degrees and their neck in a neutral position. The mean arterial pressure is kept at at least 70 mm Hg (ensuring adequate intravascular volume replacement with normal saline and using norepinephrine infusion if required), and the arterial partial pressure of oxygen (PaO_2_) is kept at at least 70 mm Hg. If ICP increases more than 20 mm Hg for more than 5 minutes without stimulation, the EVD is opened to 26 cm H_2_O and cerebrospinal fluid is drained. Opened EVDs are closed every hour to measure ICP. If an SjO_2 _monitor is present, cerebral oxygen extraction ratio is maintained at less than 40% by ensuring adequate cerebral perfusion pressure, sedation and paralysis (if required), and careful titration of arterial carbon dioxide tension to modify cerebral blood flow. Hyperthermia is avoided by using 650 mg of acetaminophen every 4 hours and cooling blankets if required to keep the core temperature at less than 37°C. Cooling measures that are more aggressive may be instituted on an individual basis to maintain an ICP of less than 20 mm Hg if the above maneuvers are insufficient. Patients are managed by fellowship-trained intensivists with backgrounds in anesthesiology, emergency medicine, internal medicine, or trauma surgery. Resident and fellow house staff is in attendance 24 hours a day.

### Statistical analysis

We used Stata 10.0 (StataCorp LP, College Station, TX, USA) to analyze our data. We described categorical data, normally distributed data, and non-normally distributed data by using proportion (percentage; 95% confidence interval, or CI), mean (standard deviation, or SD), and median (interquartile range, or IQR), respectively. We divided patients into bins on the basis of their mean 7-day hemoglobin concentrations: less than 90 g/L, 90 to 99 g/L, 100 to 109 g/L, and at least 110 g/L. These categories were inserted into the final model as indicator variables, and the range of 90 to 99 g/L was used as the referent category. Mean 7-day hemoglobin was chosen as the exposure variable since the majority of patients have reached their peak ICP by day 7 [21]. We used multivariable log-binomial regression to model the association between mean 7-day hemoglobin concentration and hospital mortality. Fractional polynomials were used to ensure the best functional form of the continuous variables of 'age' and 'GCS'. Covariates included in the final outcome model were chosen *a priori *and included the age of the patient, admission GCS score, insertion of EVD, and any RBC transfusion received. A complete-case analysis was performed, and a *P *value of less than 0.05 was considered statistically significant.

## Results

**Figure 1:**
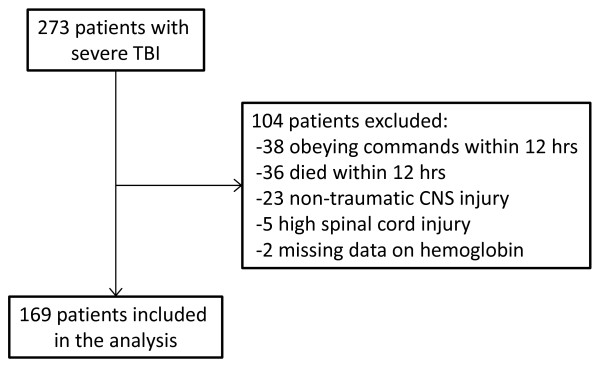
**Total patients screened and then included in the final analysis**. Reasons for exclusion are shown. CNS, central nervous system; TBI, traumatic brain injury.

In total, 273 patients with a TBI were screened for this study. One hundred four met our exclusion criteria, and 169 were included in the analysis (Figure 1). No data were missing. Patients were divided into bins on the basis of daily mean hemoglobin concentrations, and baseline characteristics are listed in Table 1. The majority of patients were male (77%), the mean age was 38 (SD 17) years, and the median best GCS score within 12 hours was 6 (IQR 5 to 7).

**Table 1:** Baseline characteristics of patients by mean 7-day hemoglobin concentration

Characteristic	Hemoglobin				*P *value
	<90 g/L ( n = 25)	90-99 g/L (n = 61)	100-109 g/L (n = 46)	≥110 g/L (n = 37)	
Age in years, mean (SD)	39 (19)	37 (18)	36 (18)	40 (15)	0.83
Male gender, number (percentage)	17 (68)	48 (80)	38 (83)	28 (76)	0.55
APACHE II score, mean (SD)	26 (5)	23 (4)	24 (5)	22 (4)	0.56
SBP <90 mm Hg, number (percentage)	6 (24)	10 (16)	9 (20)	7 (19)	0.87
PaO_2 _<70 mm Hg, number (percentage)	3 (12)	7 (11)	3 (7)	2 (5)	0.65
GCS score in 12 hours, median (IQR)	6 (5-7)	6 (6-7)	6 (5-7)	6 (6-7)	0.56
Mannitol, number (percentage)	9 (36)	31 (51)	26 (57)	19 (51)	0.14
CT of head, number (percentage)					
Traumatic subarachnoid hemorrhage	17 (68)	31 (51)	21 (46)	21 (57)	0.32
Diffuse axonal injury	12 (48)	21 (34)	9 (20)	9 (24)	0.07
Subdural hematoma	11 (44)	24 (39)	17 (37)	14 (38)	0.95
Features of increased ICP	10 (40)	31 (51)	26 (57)	20 (54)	0.60
Basal skull fracture	3 (12)	19 (31)	12 (27)	8 (22)	0.29
Craniotomy performed, number (percentage)	8 (32)	19 (31)	23 (50)	15 (41)	0.22

APACHE II, Acute Physiology and Chronic Health Evaluation II; CT, computed tomography, GCS, Glasgow Coma Scale; ICP, intracranial pressure; PaO_2_, arterial partial pressure of oxygen; SBP, systolic blood pressure; SD, standard deviation.

Clinical interventions and outcomes for the cohort are presented in Table 2. There was no difference in utilization of neurological monitoring (EVD or jugular bulb) or application of therapeutic hypothermia between levels of mean hemoglobin concentration. Overall, the median duration of intensive care was 9 (IQR 4 to 18) days, and mechanical ventilation was required for 7 (IQR 4 to 17) days. There was no difference in duration of mechanical ventilation or intensive care between groups. There were no differences in the radiographic features of TBI from computed tomography or treatment interventions among the quartiles. However, there were higher 28-day and hospital mortality rates in patients with a mean 7-day hemoglobin concentration of less than 90 g/L.

**Table 2:** Clinical interventions and outcomes in all patient quartiles

Intervention or outcome	Hemoglobin				*P *value
	<90 g/L (n = 25)	90-99 g/L (n = 61)	100-109 g/L (n = 46)	≥110 g/L (n = 37)	
Therapeutic hypothermia <35°C, number (percentage)	7 (28)	8 (13)	10 (22)	7 (19)	0.38
Therapeutic hypothermia <38°C, number (percentage)	7 (28)	22 (36)	16 (35)	12 (32)	0.92
EVD inserted, number (percentage)	14 (56)	31 (51)	29(63)	23 (62)	0.57
Jugular bulb inserted, number (percentage)	10 (40)	27 (44)	26 (56)	16 (43)	0.47
Days of EVD, median (IQR)	10 (6-16)	7 (5-12)	8 (6-12)	8 (4-12)	0.47
Days of mechanical ventilation, median (IQR)	9 (5-18)	9 (6-12)	7 (4-12)	5 (4-10)	0.29
ICU days, median (IQR)	9 (6-20)	12 (7-15)	9 (5-14)	8 (5-10)	0.44
28-day mortality, number (percentage)	10 (40)	8 (13)	9 (20)	4 (11)	0.03
Hospital mortality, number (percentage)	12 (48)	8 (13)	11 (24)	6 (16)	0.006

EVD, external ventricular drain; ICU, intensive care unit; IQR, interquartile range.

**Figure 2:**
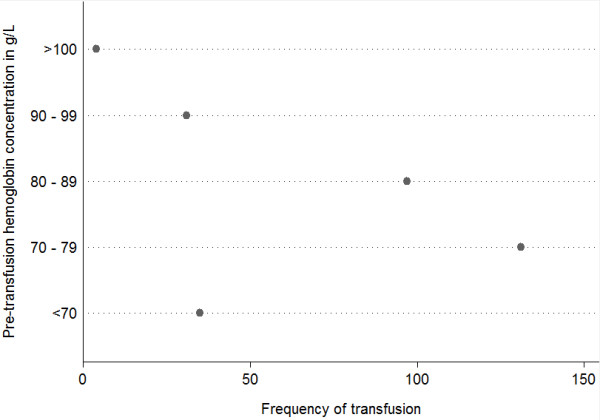
**Frequency of transfusions in each patient group according to pre-transfusion hemoglobin concentration**.

In total, 575 units of RBCs were transfused in 115 (68%) of 169 patients. Transfused patients received a median of 8 (IQR 5 to 12) units of RBCs. The median number of storage days of the RBCs was 19 (IQR 13 to 29). Two hundred seventy-seven transfusions were given in either the emergency department or operating room prior to admission to the ICU. The remaining 298 transfusions were given during the ICU stay. Pre-transfusion hemoglobin concentrations were available for packed RBCs administered in the ICU. Frequency distribution of pre-transfusion hemoglobin concentration is presented in Figure 2. Thirty-five (12%) and 131 (44%) were administered for hemoglobin concentrations of less than 70 g/L and between 70 and 79 g/L, respectively. Only 4 (1%) transfusions were administered for a hemoglobin concentration of at least 100 g/L. The median pre-transfusion hemoglobin was 79 g/L (IQR 73 to 85).

Log-binomial regression modeling the association between the mean 7-day hemoglobin and hospital mortality is presented in Table 3. Univariate analysis demonstrated that a mean 7-day hemoglobin of less than 90 g/L was associated with an increased risk of hospital mortality. This result persisted after multivariable adjustment (risk ratio (RR) 3.1, 95% CI 1.5 to 6.3, *P *= 0.002). On multivariable analysis, insertion of EVD, age, and lower admission GCS score were also associated with increased mortality. Although RBC transfusion was associated with increased hospital mortality on univariate analysis (RR 2.4, 95% CI 1.1 to 5.5, *P *= 0.03), this result did not persist in the final multivariable analysis.

**Table 3:** Log-binomial regression modeling the association between mean 7-day hemoglobin and hospital mortality

Variable	RR_unadj_	RR_adj_	95% CI	*P *value
Hemoglobin range				
<90 g/L	3.7	3.1	1.5-6.3	0.002
90-99 g/L	-	-	-	-
100-110 g/L	1.8	1.5	0.70-3.2	0.29
>110 g/L	1.2	1.2	0.49-2.7	0.75
Insertion of EVD	2.3	2.7	1.5-5.1	0.001
Age in years	1.0	1.0	1.0-1.1	<0.001
Transfusion of RBCs	2.4	1.4	0.64-2.9	0.42
Admission GCS score	0.81	0.85	0.74-0.99	0.04

CI, confidence interval; EVD, external ventricular drain; GCS, Glasgow Coma Scale; RBC, red blood cell; RR_adj_, adjusted risk ratio; RR_unadj_, unadjusted risk ratio.

## Discussion

In this retrospective cohort study of 169 patients admitted to the ICU after a severe TBI, multivariate analysis demonstrated that mean 7-day hemoglobin concentrations of less than 90 g/L were independently associated with a threefold increase in hospital mortality. Furthermore, clinicians appeared to maintain a transfusion threshold of between 70 and 90 g/L.

Anemia results in a normal physiological response to increase cardiac output and therefore cerebral blood flow. In TBI, cerebral autoregulation may be impaired and increased cerebral blood flow with anemia may result in cerebral hyperemia, which exacerbates cerebral edema and neuronal damage in TBI. Animal models of TBI have shown that normovolemic anemia results in decreased brain tissue oxygenation in comparison with a TBI without anemia [22]. Furthermore, anemia exacerbates secondary injury among rats with a TBI by enlarging cerebral contusional areas and enhancing apoptosis [22]. In humans with a TBI, transfusion of RBCs increases brain tissue oxygen [10,23]. Zygun and colleagues [23] demonstrated this improvement in brain tissue oxygenation; however, transfusion of RBCs did not into improvement in cerebral microdialysis markers of metabolism. However, microdialysis samples only a fraction of the injured brain, and it may also be that these patients were not in an oxygen delivery-dependent state [24]. Despite this beneficial physiological effect on cerebral oxygen delivery, transfusion in critically ill patients has been associated with many detrimental effects, including increased risk of nosocomial infections [13], prolonged hospital and ICU stays, and increased mortality [17]. Given these complex interactions, it is not surprising that the evidence examining the relationship between anemia and clinical outcomes is conflicting [18,25,26].

Several observational studies present often contradictory results, even within the same study. In a retrospective cohort study of patients with a severe TBI, Carlson and colleagues [19] demonstrated that transfusion and lowest measured hematocrit were associated with worse outcomes at discharge. Paradoxically, within that same study, number of days with a hematocrit of less than 30% was associated with improved outcomes as measured by GCS score and Glasgow Outcome Score (GOS). However, that analysis has many methodologic concerns - including the possibility of covariate colinearity [27], model overfitting, and misspecification - which limit the interpretability of the results. Salim and colleagues [9], in a retrospective cohort study of 1,150 TBI patients admitted to the ICU, demonstrated that transfusion, but not anemia (hemoglobin of less than 90 g/L), was associated with higher mortality. The authors did test for effect measure modification of anemia and transfusion by inserting an interaction term in the final model, which was not significant. Warner and colleagues [26] presented worse long-term functional outcomes by using the extended GOS on a linear scale with transfusion as both a linear and dichotomous variable. In a subgroup analysis of patients with a severe TBI in the TRICC trial by McIntyre and colleagues [28], the restrictive (hemoglobin of 70 to 90 g/L) and liberal (hemoglobin of 100 to 120 g/L) transfusion strategies did not differ in 30-day mortality or length of stay. However, this substudy was underpowered; only 67 patients were included between the two groups. Interestingly, a recent retrospective study by Oddo and colleagues [29] demonstrated that a hemoglobin of less than 90 g/L was associated with brain tissue oxygenation (PbtO_2_) of less than 20 mm Hg and an unfavorable outcome, which correlates with our finding that a mean 7-day hemoglobin concentration of less than 90 g/L is independently associated with a threefold increase in hospital mortality.

An interesting finding is the pre-transfusion hemoglobin levels in our study. The CRIT study, a multicenter observational cohort study of ICU patients in the US, demonstrated a mean pre-transfusion hemoglobin level of 86 g/L (SD 17), which was remarkably constant across varying types of ICUs [17]. Sena and colleagues [30] recently conducted a survey of trauma surgeons, neurosurgeons, and non-surgeon critical care physicians at level 1 trauma centers in the US to compare transfusion practices in patients with an acute severe TBI. In scenarios involving patients with normal ICPs, neurosurgeons preferred a higher transfusion threshold (83 g/L, SD 12) in comparison with trauma surgeons (75 g/L, SD 10) and non-surgeon intensivists (75 g/L, SD 8). In scenarios with elevated ICP, transfusion thresholds increased in all three groups, but the difference between neurosurgeons and the trauma surgeons and intensivists persisted (89 g/L, SD 11; 80 g/L, SD 11; and 84 g/L, SD 11, respectively) [30]. In our study, the median pre-transfusion hemoglobin of 79 g/L was consistent with transfusion thresholds of the trauma surgeons and non-intensivists in the survey by Sena and colleagues. Interestingly, our closed mixed medical-surgical ICU is staffed by fellowship-trained critical care physicians from varying subspecialty backgrounds (four anesthesiologists, two emergency medicine physicians, four internal medicine physicians, and one trauma surgeon) but does not include a neurosurgeon intensivist. However, the comparison of our results with the study by Sena and colleagues should be interpreted with caution as the latter represents survey data rather than actual transfusion thresholds from a clinical study.

A major limitation of all available clinical studies, ours included, is the inadequate consideration of transfusion in the analysis. Appropriate adjustment for transfusion may help reduce bias between anemia and outcome but may hide an important relationship across subgroups. For example, patients with the same hemoglobin concentration of 90 g/L may have very different outcomes, depending on whether they receive an RBC transfusion or not. Thus, rather than statistical adjustment of transfusion, it may be more informative to look for effect measure modification between hemoglobin levels and RBC transfusion. Because of our small sample size, we were unable to test for these important subgroup effects. Furthermore, we considered transfusion a time-insenstive dichotomous variable in only the final multivariable model, which fails to account for the important interaction between time, transfusion, and hemoglobin concentration. Additional limitations of our study need to be addressed. By using indicator variables that allow a non-linear relationship of hemoglobin concentration to mortality in our model, we have tried to avoid model misspecification. It is possible that residual confounding may occur within strata of hemoglobin concentrations. More importantly, it is unclear how best to measure 'exposure' to hemoglobin levels. *A priori*, we chose mean 7-day hemoglobin concentration because the majority of patients have achieved their peak ICP by that time period. Other observational studies have used a variety of similar approaches, including number of days with a hematocrit of less than 30% and lowest mean hematocrit [19], a hemoglobin of less than 90 g/L for three consecutive measurements [9], and lowest hematocrit level [31]. All of these approaches have limitations that may result in exposure misclassification. In addition, the decision to transfuse would be subject to strong confounding by indication [32]. Thus, even with regression adjustment for transfusion, there would be residual or unmeasured confounding, which remains an alternative explanation for our results. In keeping with many of the aforementioned studies on TBI, we do not have outcome data on functional status, which is arguably more important in this population. These important limitations notwithstanding, this study should be used to guide further hypotheses and study designs to more formally assess the relationship between anemia, transfusion, and outcomes in patients with a TBI.

## Conclusions

We have demonstrated that a mean 7-day hemoglobin concentration of less than 90 g/L may be harmful in patients with a severe TBI. Large well-controlled observational studies that are powered to examine important subgroup effects (for example, RBC transfusion by hemoglobin concentration) and time-varying covariates (for example, ICP) are warranted to characterize both hemoglobin concentrations and transfusion thresholds to guide clinicians managing patients with a TBI.

## Key messages

• Definitive data on transfusion thresholds and anemia management in TBI are lacking.

• A hemoglobin level of less than 90 g/L is associated with a threefold increase in hospital mortality.

• Large and well-designed multicenter observational studies and randomized control trials are needed to further elucidate transfusion thresholds for anemia in patients with a TBI.

## Abbreviations

CI: confidence interval; EVD: external ventricular drain; GCS: Glasgow Coma Scale; GOS: Glasgow Outcome Score; ICP: intracranial pressure; ICU: intensive care unit; IQR: interquartile range; RBC: red blood cell; RR: risk ratio; SD: standard deviation; SjO_2_: jugular venous oximetry; TBI: traumatic brain injury; TRICC: Transfusion Requirements in Critical Care; VGH: Vancouver General Hospital.

## Competing interests

The authors declare that they have no competing interests.

## Authors' contributions

MSS conducted the literature search and contributed to data analysis and interpretation, writing, and manuscript preparation. NM contributed to the study design. DRC contributed to the study design, data analysis and interpretation, and manuscript preparation. WRH contributed to data interpretation and manuscript preparation. DEG contributed to the study design; data collection, analysis, and interpretation; writing; and manuscript preparation. All authors read and approved the final manuscript.
